# Robotic versus Video-Assisted Thoracic Surgery for Lung Cancer: Short-Term Outcomes of a Propensity Matched Analysis

**DOI:** 10.3390/cancers15082391

**Published:** 2023-04-21

**Authors:** Savvas Lampridis, Alessandro Maraschi, Corinne Le Reun, Tom Routledge, Andrea Billè

**Affiliations:** 1 Department of Thoracic Surgery, Guy’s Hospital, Guy’s and St Thomas’ NHS Foundation Trust, London SE1 9RT, UK; 2 Independent Biostatistician, 97180 Sainte-Anne, Guadeloupe, France

**Keywords:** lobectomy, lung cancer, minimally invasive surgery, robotic surgery, segmentectomy, video-assisted thoracic surgery (VATS)

## Abstract

**Simple Summary:**

This study compared two types of surgery for lung cancer: robot-assisted thoracic surgery (RATS) and video-assisted thoracic surgery (VATS). The researchers looked at the results of 613 patients with similar characteristics who had surgery between 2015 and 2020. They found that RATS took longer than VATS, but there was less bleeding with RATS. The two types of surgery were similar in terms of other outcomes, such as complications after surgery, duration of hospital stay, readmissions, and deaths. More research is needed to see if one surgical approach has significant benefits over the other.

**Abstract:**

Robot-assisted thoracic surgery (RATS) has gained popularity for the treatment of lung cancer, but its quality outcome measures are still being evaluated. The purpose of this study was to compare the perioperative outcomes of lung cancer resection using RATS versus video-assisted thoracic surgery (VATS). To achieve this aim, we conducted a retrospective analysis of consecutive patients who underwent lung cancer surgery between July 2015 and December 2020. A propensity-matched analysis was performed based on patients’ performance status, forced expiratory volume in 1 s% of predicted, diffusing capacity of the lungs for carbon monoxide% of predicted, and surgical procedure (lobectomy or segmentectomy). Following propensity matching, a total of 613 patients were included in the analysis, of which 328 underwent RATS, and 285 underwent VATS, with satisfactory performance indicators. The results of the analysis indicated that RATS had a significantly longer operating time than VATS (132.4 ± 37.3 versus 122.4 ± 27.7 min; mean difference of 10 min 95% CI [confidence interval], 4.2 to 15.9 min; *p* = 0.001). On the other hand, VATS had a significantly higher estimated blood loss compared to RATS (169.7 ± 237.2 versus 82.2 ± 195.4 mL; mean difference of 87.5 mL; 95% CI, 48.1 to 126.8 mL; *p* < 0.001). However, there were no significant differences between the groups in terms of the duration of chest tubes, length of hospital stay, low- and high-grade complications, as well as readmissions and mortality within 30 days after surgery. Moreover, the number of dissected lymph-node stations was significantly higher with VATS than RATS (5.9 ± 1.5 versus 4.8 ± 2.2; mean difference of 1.2; 95% CI, 0.8 to 1.5; *p* = 0.001). Nonetheless, the percentage of patients who were upstaged after histopathological analysis of the resected lymph nodes was similar between the two groups. In conclusion, RATS and VATS yielded comparable results for most of the short-term outcomes assessed. Further research is needed to validate the implementation of RATS and identify its potential benefits over VATS.

## 1. Introduction

Surgical resection is the standard of care for patients with early stage non-small-cell lung cancer (NSCLC) [1]. Historically, surgery for lung cancer was performed via thoracotomy with rib spreading. Advances in medical technology, however, led to the introduction of video-assisted thoracoscopic surgery (VATS) in the early 1990s. Since then, VATS has been increasingly used as an alternative to open surgery, and it currently represents the preferred approach for lung cancer resection in most institutions. The main reasons for this paradigm shift include reduced postoperative morbidity, less pain, shorter hospitalization, better recovery of physical function, and equivalent, if not superior, oncological outcomes with VATS compared to open thoracotomy [2,3,4,5,6,7]. Despite these improved results, VATS continues to be characterized by certain technical constraints, including compromised dexterity, limited degrees of motion, fulcrum effect, and the amplification of physiologic tremors [8]. As an answer to the limitations of VATS, robot-assisted thoracic surgery (RATS) has recently come to the forefront as a new platform for lung cancer surgery. In a period of only 4 years, between 2011 and 2015, the centers performing robot-assisted lobectomies increased 90% worldwide [9]. In the USA only, the percentage of minimally invasive lobectomies performed with RATS rose from 0.3% in 2009 to 16.2% in 2013, while the number of centers reporting experience with robot-assisted lobectomy also increased [10]. Despite its continuous growth, however, the widespread adoption of RATS has been mainly hampered by its higher cost compared to VATS [11,12].

Outcome data comparing RATS with VATS for lung cancer resection remain sparse and are challenging to interpret, especially in the absence of large prospective randomized trials. Available data regarding long-term oncological outcomes following minimally invasive thoracic surgery for lung cancer suggest oncologic equivalence between RATS and VATS at high-volume centers [13,14]. Therefore, quality outcome measures, such as perioperative complications, length of hospital stay, and lymph nodal upstaging, may play a significant role in the choice of surgical approach. Nevertheless, results from different studies are mixed and conflicting. For instance, early experience with robot-assisted lobectomy was associated with a higher rate of intraoperative injury and bleeding, as well as longer operating times, compared to VATS [12,15]. However, subsequent studies challenged these findings, showing no significant difference in complication rates between the two approaches, as well as shorter hospital stays with RATS [10,16,17]. Similarly, one study demonstrated that VATS was superior compared to RATS regarding lymph-node yield and upstaging [18], while another study indicated comparable results [19]. Finally, it should be noted that comparisons between RATS and VATS are hindered by the fact that technical aspects are seldom specified, while it may also be unclear where on the learning curve surgeons exist for different studies.

With robot-assisted lung resections performed at more institutions and an increasing number of surgeons past their learning curves, it becomes evident that an updated comparative analysis between RATS and VATS is timely and needed. The aim of this study was to compare the perioperative outcomes of lung cancer resection with RATS versus VATS.

## 2. Materials and Methods

### 2.1. Study Design and Patient Selection

We conducted a retrospective, comparative cohort study of consecutive adult patients who underwent lung cancer resection with either RATS or VATS at Guy’s Hospital, London, UK, between July 2015 and December 2020. During the first three years of the study, all patients underwent VATS. Following the acquisition of a robotic surgical system, we transitioned to performing RATS for the subsequent cases. The study conformed to the ethical principles defined in the Declaration of Helsinki of 1964 and all subsequent revisions, and it was approved by the relevant committee of our institution as a service evaluation project (Number 13197). Informed patient consent was not required due to the retrospective nature of the study.

Patients were included in the study if a diagnosis of lung cancer was confirmed after histopathological analysis of the resected specimens, regardless of the extent of resection or stage of the disease. Conversely, patients were excluded from the study if they were younger than 18 years of age or if they underwent lung resection for any other indication apart from lung cancer.

### 2.2. Patient Management

Preoperative evaluation of all patients followed the clinical practice guidelines published by the National Cancer Comprehensive Network [20], American College of Chest Physicians [21], and European Respiratory Society/European Society of Thoracic Surgeons [22]. Perioperatively, all patients were treated uniformly according to a predefined departmental protocol that was based on recommendations of the Enhanced Recovery After Surgery Society and the European Society of Thoracic Surgeons [23].

### 2.3. Operative Technique

All robot-assisted procedures were performed with the da Vinci Xi surgical system (Intuitive Surgical, Sunnyvale, CA, USA). In general, lobectomies were performed based on the technique described by Veronesi et al. [24], and segmentectomies were performed based on the method introduced by Pardolesi et al. [25]. VATS was carried out using 3 ports. Specifically, a 3 cm utility incision was made at the 4th or 5th intercostal space in the anterior axillary line, a 1 cm port was placed at the 7th intercostal space in the midaxillary line for a 30-degree thoracoscope, and a further 1 cm utility port was inserted at the 7th intercostal space in the posterior axillary line. Pulmonary vessels and bronchi were divided with mechanical staplers (SureForm [Intuitive] in RATS and Endo GIA [Medtronic, Dublin, Ireland] in VATS), and the specimen was retrieved with an Endo Catch pouch (Medtronic). Regardless of approach, the dissection of lymph nodes was generally undertaken from stations 2R, 4R, 7, 10R and 11R for right-sided tumors, and stations 5, 6, 7, 10L and 11L for left-sided tumors, with the inclusion of lymph nodes from station 9 for lower lobe tumors. Peripheral lung lesions without a preoperative tissue diagnosis were managed with wedge resection and frozen section analysis, which was followed by anatomic lung resection in the case of lung cancer. All procedures were performed by two surgeons, both of whom had more than 10 years of experience in VATS and were at the beginning of the learning curve with RATS.

### 2.4. Variables and Outcomes

We reviewed electronic medical records and a prospectively maintained departmental database to collect data on the following variables: sex, age, body-mass index, performance status, comorbidities, smoking history, pulmonary function tests, pathologic diagnosis, neoadjuvant therapy, as well as surgical approach and procedure. The outcomes of interest included operating time, estimated blood loss, transfusion of blood products, duration of chest tube, length of hospital stay, postoperative morbidity, as well as readmission and mortality within 30 days of surgery. Staging was reported according to the eighth edition of the lung cancer stage classification [26]. Postoperative complications were graded according to the revised Clavien–Dindo classification [27]. Grade II or lower complications were defined as low grade, whereas grade III or greater complications were defined as high grade. Prolonged air leak was defined as air leak lasting more than 7 days [28].

### 2.5. Statistical Analysis

Data were summarized with frequency and percentage for categorical variables and mean ± standard deviation for continuous variables. Comparisons of data between the groups were made using the t-test for continuous variables and Fisher’s exact test or the chi-squared test for categorical variables (for 2 × 2 or larger contingency tables, respectively). To minimize selection bias due to the nonrandom allocation of treatments, the patients were matched on covariates that have been generally accepted as factors associated with the outcomes of interest. Patients were matched on the following characteristics: performance status, forced expiratory volume in 1 s (FEV1)% of predicted, diffusing capacity of the lungs for carbon monoxide (DLCO)% of predicted, and surgical procedure (lobectomy or segmentectomy). The propensity score used for matching was created using a logit model. Matching was performed using the nearest neighbor approach, matching each RATS patient to 2 VATS patients. An initial matching using only the nearest neighbor was initially tested, but the matching proved more satisfactory with two. The balance of covariates after the matching was checked with standardized differences and a summary of the mean and median bias across all covariates before and after matching, as well as Rubin’s B indicator (i.e., the absolute standardized difference of the means of the linear index of the propensity scores between the groups) and Rubin’s R indicator (i.e., the ratio of the variances of the propensity score index in the groups). Ideally, the overall bias should be below 5, Rubin’s B less than 25, and Rubin’s R between 0.5 and 2. Treatment effects comparing RATS and VATS were reported as mean differences for continuous outcomes and risk differences for categorical outcomes, with a 95% confidence interval and *p* value. All *p* values were two-sided, with statistical significance accepted as *p* < 0.05.

## 3. Results

A total of 746 patients were included in the study. Of those, 366 (49.1%) patients underwent RATS, and 380 (50.9%) patients underwent VATS. The characteristics of the patients are detailed in Table 1. Of the performed procedures, 496 (66.5%) were lobectomies, 143 (19.2%) were segmentectomies, 95 (12.7%) were wedge resections, and 12 (1.6%) were bilobectomies. The type of procedures was significantly different between the groups, with more lobectomies performed with RATS (76% versus 57.4%; *p* < 0.001) and more wedge resections with VATS (22.4% versus 2.7%; *p* < 0.001). Conversion to open thoracotomy was performed in 16 (4.4%) patients undergoing RATS and 11 (2.9%) patients undergoing VATS (*p* = 0.280).

A total of 613 patients were selected and analyzed following propensity matching. Of those, 328 patients underwent RATS and 285 underwent VATS. Prior to propensity matching, the characteristics of both groups were globally similar; however, some imbalances were observed in the DLCO% of predicted and the type of surgical procedure. These were corrected by the matching method, and all the performance indicators were satisfactory. In particular, the overall mean and median bias was 2.3% and 2.4%, respectively, Rubin’s B was 5, and Rubin’s R was 0.83. Table 2 displays the matching characteristics of the groups.

The perioperative outcomes are presented in Table 3. The operating time was significantly longer with RATS than VATS, with a mean difference of 10 min (132.4 ± 37.3 versus 122.4 ± 27.7 min; 95% CI [confidence interval], 4.2 to 15.9 min; *p* = 0.001). The estimated blood loss was significantly higher with VATS compared to RATS (169.7 ± 237.2 versus 82.2 ± 195.4 mL; mean difference of 87.5 mL; 95% CI, 48.1 to 126.8 mL; *p* < 0.001). Regarding postoperative outcomes, there were no significant differences between the groups for either low- or high-grade complications. In particular, low-grade complications were observed in 28.0% of the patients in the RATS group versus 24.2% in the VATS group (*p* = 0.340), while high-grade complications were documented in 7.0% in the RATS group and 6.4% in the VATS group (*p* = 0.794). However, within the subgroup of different complications, subcutaneous emphysema resulting from air leak was significantly higher in the VATS group (3.2% versus 0.3%; risk difference of 2.9%; 95% CI, 0.3% to 5.5%; *p* = 0.030). There were no significant differences between the groups regarding the duration of chest tubes, length of hospital stay, as well as readmissions and mortality within 30 days after surgery. The number of dissected lymph-node stations was significantly higher with VATS than RATS (5.9 ± 1.5 versus 4.8 ± 2.2; mean difference of 1.2; 95% CI, 0.8 to 1.5; *p* = 0.001). However, the percentage of patients who were upstaged after histopathological analysis of the resected lymph nodes was similar between the groups.

## 4. Discussion

The ideal surgical approach for the treatment of lung cancer should minimize perioperative complications, facilitate enhanced recovery, promote expedient return to daily living activities, and provide sound pathological outcomes, including complete resection of the disease with adequate lymphadenectomy. Although these objectives are widely accepted by surgeons, the approach of choice remains controversial. In this study, we compared VATS, which has been established as the preferred approach in most centers, with RATS, which has recently emerged as a valid alternative, regarding short-term outcomes after anatomic lung resection for cancer.

Based on the matched patient cohorts, there does not appear to be a significant difference between RATS and VATS for most of the outcomes of interest. In particular, there was no difference in conversion to an open approach, transfusion of blood products, pathologic lymph-node upstaging, duration of chest tubes, length of hospital stay, low- and high-grade complications, hospital readmissions, and mortality. In contrast, significant differences were observed in the operating time, estimated blood loss, and the number of dissected nodal stations.

Regarding the operating time, RATS lasted longer than VATS by 10 min on average (95% CI, 4.2 to 15.9 min; *p* = 0.001). Nonetheless, it should be mentioned that the operating time of RATS includes the learning curve with this approach. Therefore, this result should be interpreted with caution, considering that operating time decreases with experience [8]. Indeed, in a retrospective study investigating the learning curve of RATS for lobectomy, which included 208 consecutive patients with primary lung cancer, the total surgical time decreased from 197 ± 28 to 141 ± 29 min (*p* < 0.001) after surgical proficiency was achieved [29]. Similarly, in a study comparing the learning curve of RATS and VATS for lobectomy in patients with NSCLC using cumulative sum analysis, the mean operating time of RATS decreased from 196 ± 71 min in the first 45 cases to 155 ± 39 min in the following 30 cases (*p* = 0.001) [30]. Interestingly, the mean operating time of VATS decreased from 183 ± 51 min in the first 53 cases to 143 ± 32 min in the following 22 cases. In the present study, the mean operating time with VATS was 122 ± 28 min, including only late experience with this approach, while with RATS was 132 ± 37 min, including both late and early experience. In a meta-analysis of 13 studies comparing RATS (*n* = 3995) with VATS (*n* = 4211) for lobectomy or segmentectomy in patients with NSCLC, there was no significant difference in operating time between the groups (weighted mean difference, −0.79 min; 95% CI, −15.65 to 14.06 min; *p* = 0.920; I2 = 97%) [31].

The estimated blood loss was another intraoperative outcome that differed significantly between the groups. The blood loss associated with RATS was less by 87.5 mL compared to VATS (95% CI, 48.1 to 126.8 mL; *p* < 0.001). This result is consistent with those reported in previous studies comparing RATS with VATS for the surgical resection of NSCLC. In a pooled analysis of the data from seven studies, RATS (*n* = 385) was associated with significantly less blood loss than VATS (*n* = 483; weighted mean difference, 50 mL; 95% CI, 10 to 90 mL; *p* = 0.010; I2 = 93%). A plausible explanation for the recorded differences in the estimated blood loss between RATS and VATS may lie in the technical advantages provided by the robotic platform. The three-dimensional, magnified field of view, the elimination of tremor, and the more flexible equipment offered by RATS can allow surgeons to better identify anatomical relationships and control bleeding that occurs during the dissection of hilar and mediastinal lymph nodes, which represents an important source of blood loss during lung cancer surgery [32].

Despite these potential advantages in surgical technique with RATS, VATS was associated with a significantly more extensive lymphadenectomy in our study. The mean difference of dissected nodal stations between the groups was 1.2 (95% CI, 0.8 to 1.5; *p* < 0.001). Although statistically significant, this difference may not be as significant clinically since it did not translate into a difference in pathologic lymph-node upstaging. In both groups, 11.9% of the patients were upstaged from a clinical stage of N0 or N1 to a higher pathologic N stage. Conversely, a meta-analysis of pooled results from eight studies comparing RATS (*n* = 997) with VATS (*n* = 1144) for anatomic lung resection showed that the number of dissected nodal stations was higher with RATS than VATS (weighted mean difference, 0.51; 95% CI, 0.15 to 0.86; *p* = 0.005; I2 = 86%) [31]. This thoroughness in lymphadenectomy with RATS was attributed to an improved dissection of deep tissues in complex anatomical locations, such as the mediastinum and pulmonary hila [31], and it has been reflected in pathologic lymph-node upstaging in multiple studies [33,34]. In a multicenter, retrospective study of 302 patients with clinical stage I NSCLC who underwent RATS for lobectomy or segmentectomy, pathologic nodal upstaging occurred in 33 (10.9%) patients, with a rate of hilar (pN1) upstaging of 6.6% and a rate of mediastinal (pN2) upstaging of 4.3% [33]. These results are comparable to those reported in retrospective studies comparing VATS with open thoracotomy [35,36,37]. In a study of 4394 patients with clinical stage I NSCLC who underwent resection by VATS, as identified from the General Thoracic Surgery Database of the Society of Thoracic Surgeons, the rate of overall pathologic lymph-node upstaging achieved by VATS was 11.6%. A similar rate (11.9%) was also identified in an analysis of the Danish Lung Cancer Registry regarding pathologic nodal upstaging after resection of clinical stage I NSCLC by VATS [36]. In a propensity-score-adjusted comparison of pathologic nodal upstaging that included 911 patients, RATS was associated with a higher overall rate of upstaging than VATS (16.2% versus 12.3%; *p* = 0.03) [34]. A thorough assessment of hilar and mediastinal lymph nodes is crucial to ensure the detection of occult metastases, and thus the accurate staging of lung cancer.

There were no significant differences between the two surgical approaches in terms of postoperative mortality and overall morbidity. Regarding the latter, however, a significantly higher proportion of patients undergoing VATS experienced subcutaneous emphysema compared to those who underwent RATS (3.2% versus 0.3%; *p* = 0.03). Subcutaneous emphysema is a postoperative complication that can lead to increased patient distress, empyema, and prolonged hospitalization [38]. A strong risk factor for postoperative air leak and subcutaneous emphysema is the presence of adhesions between the visceral pleura and surrounding structures [39,40]. Arguably, the seven degrees of freedom provided by the robotic articulating dissecting instruments may allow for a more precise division of pulmonary adhesions in the recesses and at the apex of the chest cavity, which could translate to a lower percentage of patients with postoperative air leak and associated subcutaneous emphysema. Interestingly, in a multicenter, randomized controlled trial of patients who underwent surgery for clinical stage N2 NSCLC, RATS achieved similar rates of postoperative subcutaneous emphysema when compared with an open approach (3.9% versus 2.8%, respectively; *p* > 0.99) [41].

An important factor that should be considered when discussing the results of the present study is the potential impact of the COVID-19 pandemic. Indeed, most of the robotic pulmonary resections included in the analysis were performed during the pandemic, while no VATS was undertaken during this period. The COVID-19 pandemic has negatively affected outcomes in thoracic surgery, with a modification of the standard protocols for early-stage NSCLC and a reduction in the number of pulmonary resections [42,43,44,45]. In a multicenter cohort study that was conducted across all thoracic surgical units in London and included 352 patients undergoing anatomic lung resection for NSCLC during the COVID-19 pandemic, there was an increased rate of significant complications (grade III or IV according to the Clavien–Dindo classification), as well as of hospital readmissions [46]. These findings indicate that the COVID-19 pandemic may have negatively affected the postoperative short-term outcomes in the RATS group of our study.

The present study is characterized by certain limitations. Firstly, although patient matching minimized selection bias among the groups, the retrospective nature of the study may have still influenced the results. Furthermore, matching for other variables could have been performed, but we selected clinically meaningful characteristics available at the time of surgery. Moreover, another bias may have been introduced by the selection of surgical approach based on the surgeon’s expertise and patient’s preferences. Finally, we did not compare the average hospital costs per patient between the two approaches, which may impact clinical decisions in these times of increasing healthcare expenditure. A comprehensive cost comparison between the two surgical approaches is available in reference [47].

## 5. Conclusions

In a propensity matched analysis of patients with lung cancer who underwent anatomic pulmonary resection, the results of RATS and VATS were found to be similar in most of the short-term outcomes of interest. Nonetheless, statistically significant differences were noted in operating time, estimated blood loss, and number of dissected nodal stations. These differences, however, should be interpreted with caution, since they may not be clinically meaningful or could be influenced by the learning curve associated with RATS. Considering the increasing number of patients undergoing RATS for lung cancer and the rising number of companies introducing robotic platforms into the market, ongoing research is necessary to further validate the implementation of this technology and identify its potential benefits over conventional surgical approaches.

## Figures and Tables

**Table 1:** Patient characteristics ^1^.

Variable	RATS (*n* = 366)	VATS (*n* = 380)	*p* Value
Age, years	69.7 ± 9.8	69.1 ± 9.9	0.373
Sex, *n* (%)			0.269
Female	234 (63.9)	228 (60.0)	
Male	132 (36.1)	152 (40.0)	
BMI, kg/m ^2^	27.5 ± 5.6	27.1 ± 5.3	0.289
Smoking status, *n* (%)			0.166
Non-smoker	81 (22.1)	63 (16.6)	
Ex-smoker	219 (59.8)	254 (66.8)	
Smoker	62 (16.9)	63 (16.6)	
Unknown	3 (0.8)		
Pulmonary function			
FEV1% of predicted	91.9 ± 21.2	92.3 ± 22.3	0.790
FVC% of predicted	108.4 ± 20.8	108.9 ± 21.3	0.742
DLCO% of predicted	76.5 ± 20.1	72.2 ± 18.8	0.003
Comorbidities, *n* (%)			
Atrial fibrillation	30 (8.2)	26 (6.8)	<0.001
Bronchiectasis	6 (1.6)		0.012
Cerebrovascular accident	16 (4.4)	13 (3.4)	0.502
Chronic kidney disease	14 (3.8)	19 (5.0)	0.435
COPD	80 (21.9)	92 (24.2)	0.446
Coronary artery disease	47 (12.8)	51 (13.4)	0.815
Interstitial lung disease	1 (0.3)	5 (1.3)	0.111
Peripheral vascular disease	3 (0.8)	3 (0.8)	0.963
Previous other cancer	147 (40.2)	125 (32.9)	0.039
Previous primary lung cancer	18 (4.9)	19 (5.0)	0.959
Pulmonary hypertension	2 (0.5)	1 (0.3)	0.541
Charlson Comorbidity Index, ^2^ median (range)	6 (0–19)	6 (0–18)	0.977
Induction chemotherapy, *n* (%)	8 (2.2)	10 (2.6)	0.692
Histopathology, *n* (%)			0.820
Adenocarcinoma	226 (61.7)	217 (57.1)	
Squamous cell carcinoma	49 (13.4)	63 (16.6)	
Carcinoid tumor	34 (9.3)	24 (6.3%)	
Other	57 (15.6)	76 (20)	
Clinical stage, *n* (%)			
0	2 (0.5)	1 (0.3)	
IA1	23 (6.3)	16 (4.2)	
IA2	83 (22.7)	98 (25.8)	
IA3	65 (17.8)	43 (11.3)	
IB	57 (15.6)	64 (16.8)	
IIA	19 (5.2)	20 (5.3)	
IIB	37 (10.1)	36 (9.5)	
IIIA	24 (6.6)	32 (8.4)	
IIIB	5 (1.4)	7 (1.8)	
IIIC			
IVA	1 (0.3)	1 (0.3)	
IVB		1 (0.3)	
Unknown	50 (13.7)	61 (16.1)	

^1^ Plus–minus values are means ± standard deviations. ^2^ The Charlson Comorbidity Index quantifies an individual’s burden of disease, with scores ranging from 0 to 37; the higher the score, the more likely the predicted outcome will result in mortality or higher resource use. COPD, chronic obstructive pulmonary disease; DLCO, diffusing capacity of the lungs for carbon monoxide; FEV1, forced expiratory volume in 1 s; FVC, forced vital capacity.

**Table 2:** Matching characteristics of the patients.

Variable	All Patients		Propensity-Matched Patients	
	RATS	VATS	RATS	VATS
ECOG performance status ^1^,%				
Grade 0	31.4	31.3	31.4	31.3
Grade 1	49.7	49.8	49.7	50.9
Grade 2	18.9	18.9	18.9	17.8
FEV1% of predicted, mean	92.2	92.5	92.2	91.4
DLCO% of predicted, mean	76.6	72.7	76.6	76.2
Procedure,%				
Lobectomy	79.0	75.1	79.0	79.1
Segmentectomy	21.0	24.9	21.0	20.9

^1^ The ECOG performance status grades an individual’s performance status from 0 to 4, with higher grades indicating greater limitation in a patient’s daily living activities. ECOG, Eastern Cooperative Oncology Group; DLCO, diffusing capacity of the lungs for carbon monoxide; FEV1, forced expiratory volume in 1 s.

**Table 3:** Surgical outcomes after propensity matching.

Outcome	RATS	VATS	Difference RATS vs. VATS (95% CI)	*p* Value
Operating time, min	132.4 ± 37.3	122.4 ± 27.7	10.0 (4.2, 15.9)	0.001
Estimated blood loss, ml	82.2 ± 195.4	169.7 ± 237.2	−87.5 (−126.8, −48.1)	<0.001
Transfusion of blood products,%	1.8	4.9	−3.1 (−7.3, 1.2)	0.161
Conversion to thoracotomy,%	4.3	3.2	1.1 (−2.5, 4.7)	0.562
Complications,%				
Atelectasis requiring bronchoscopy	5.5	2.7	2.7 (−0.4, 5.9)	0.092
Atrial fibrillation	8.5	6.1	2.4 (−1.6, 6.5)	0.238
Bleeding requiring reoperation	1.2	0.8		0.582
Empyema	2.1	0.9	1.2 (−0.8, 3.3)	0.245
Pneumonia	11.0	9.6	1.4 (−3.7, 6.5)	0.599
Prolonged air leak ^1^	6.7	7.9	−1.2 (−5.7, 3.2)	0.590
Pneumothorax requiring drain insertion	3.1	2.3	0.8 (−2.4, 3.9)	0.638
Subcutaneous emphysema	0.3	3.2	−2.9 (−5.5, −0.3)	0.030
Low-grade complications ^2^,%	28.0	24.2	3.8 (−4.0, 11.6)	0.340
High-grade complications ^2^,%	7.0	6.4	0.6 (−4.0, 5.2)	0.794
ICU admission (unplanned),%	8.8	12.0	−3.2 (−8.9, 2.5)	0.269
Reoperation,%	2.1	1.2	0.9 (−1.1, 3.0)	0.385
Chest tube duration, days	3.5 ± 4.4	3.5 ± 4.6	0.03 (−0.8, 0.9)	0.937
Length of hospital stay, days,%	6.8 ± 8.6	6.1 ± 5.3	0.7 (−0.4, 1.9)	0.223
Readmission within 30 days,%	6.7	4.9	1.8 (−2.8, 6.5)	0.443
Mortality within 30 days,%	0.8	0.8	0 (−1.6, 1.6)	>0.99
Lymph node stations dissected	4.8 ± 2.2	5.9 ± 1.5	−1.2 (−1.5, −0.8)	<0.001
Lymph node upstaging,%				
cN0 to pN1	6.3	4.5	1.7 (−3.0, 6.5)	0.470
cN0 to pN2	4.5	6.3	−1.7 (−5.6, 2.2)	0.380
cN1 to pN2	1.0	1.0	0.0 (−1.7, 1.7)	>0.99

^1^ Prolonged air leak was defined as an air leak lasting longer than 7 days after the operation. ^2^ The Clavien–Dindo classification categorizes complications as grade I for any deviation from the normal postoperative course, grade II for those requiring pharmacological treatment, grade III for those requiring surgical, endoscopic, or radiological intervention (IIIa not under general anesthesia, IIIb under general anesthesia), and grade IV for those requiring management in the intensive care unit (IVa for single-organ failure, IVb for multiorgan failure); grade V denotes the death of a patient. Grade II or lower complications were defined as low grade; grade III or greater complications were defined as high grade. CI, confidence interval; ICU, Intensive care unit.

## Data Availability

The data presented in this study are available on request from the corresponding author.
